# Clinical contributions of exhaled volatile organic compounds in the diagnosis of lung cancer

**DOI:** 10.1371/journal.pone.0174802

**Published:** 2017-04-06

**Authors:** Tsuyoshi Oguma, Takashi Nagaoka, Muneshige Kurahashi, Naofumi Kobayashi, Shinji Yamamori, Chizuko Tsuji, Hiroto Takiguchi, Kyoko Niimi, Hiromi Tomomatsu, Katsuyoshi Tomomatsu, Naoki Hayama, Takuya Aoki, Tetsuya Urano, Kazushige Magatani, Sunao Takeda, Tadashi Abe, Koichiro Asano

**Affiliations:** 1 Division of Pulmonary Medicine, Department of Medicine, Tokai University School of Medicine, Kanagawa, Japan; 2 Research Institute for Science and Engineering, Waseda University, Tokyo, Japan; 3 Ogino Memorial Laboratory, Nihon Kohden Corporation, Tokyo, Japan; 4 Department of Physiology, Tokai University School of Medicine, Kanagawa, Japan; 5 Department of Electrical and Electronic Engineering, Tokai University School of Engineering, Kanagawa, Japan; National and Kapodistrian University of Athens, GREECE

## Abstract

**Background:**

Exhaled volatile organic compounds (VOC) are being considered as biomarkers for various lungs diseases, including cancer. However, the accurate measurement of extremely low concentrations of VOC in expired air is technically challenging. We evaluated the clinical contribution of exhaled VOC measured with a new, double cold-trap method in the diagnosis of lung cancer.

**Methods:**

Breath samples were collected from 116 patients with histologically confirmed lung cancer and 37 healthy volunteers (controls) after inspiration of purified air, synthesized through a cold-trap system. The exhaled VOC, trapped in the same system, were heat extracted. We analyzed 14 VOC with gas chromatography.

**Results:**

The concentrations of exhaled cyclohexane and xylene were significantly higher in patients with lung cancer than in controls (*p* = 0.002 and 0.0001, respectively), increased significantly with the progression of the clinical stage of cancer (both *p* < 0.001), and decreased significantly after successful treatment of 6 patients with small cell lung cancer (p = 0.06 and 0.03, respectively).

**Conclusion:**

Measurements of exhaled VOCs by a double cold-trap method may help diagnose lung cancer and monitor its progression and regression.

## Introduction

Over 200 and 60 x 10^3^ new cases of lung cancer are diagnosed each year in the United States and in Japan, respectively. It is the leading cause of cancer-related deaths and accounts for over 1/4 of all cancer fatalities in men and women [1,2]. Because of the poor prognosis of this disease when advanced, reliable screening methods to detect lung cancers at an early stage are highly desirable. The yield of annual chest radiographs to screen for lung cancer is low. Thoracic computed tomography scans lower the mortality due to lung cancer in high risk patients [3]. However, they are limited by insufficient financial resources and excessive exposure to radiation. Therefore, a reliable, inexpensive and less invasive approach for the screening of lung cancer needs to be developed.

The expired human gas contains over 200 volatile organic compounds (VOC), such as aromatic hydrocarbons, alicyclic hydrocarbons, and chain hydrocarbons. Therefore, VOC in exhaled air are being considered as possible biomarkers of various pulmonary diseases, including lung cancer [4]. However accurate measurements of extremely low concentrations of VOC in expired air have been technically challenging, without being contaminated by atmospheric VOC. Several VOC sensors of exhaled air have been developed. eNose, a gas sensor array, is an easy-to-use, commercially available system, which, however, does not measure the concentrations of specific VOC [5]. A cold-trap method to concentrate human expired gas, combined with gas chromatography, is another approach. We have developed a modified “double” cold-trap method that can measure trace amounts of exhaled VOC, and report the contribution of their measurements to the management of patients presenting with lung cancers.

## Methods

### Study participants and data collection

Between July 2012 and November 2013, we collected breath samples from 116 patients with histologically confirmed lung cancers and from 37 healthy volunteers at Tokai University Hospital. The healthy volunteers had no history of major pulmonary disease and were free from disease in any other organ. Patients with lung cancer presenting with an Eastern Cooperative Oncology Group performance status ≥2, or with MRC scores ≥3 were excluded from this study [6]. The patients’ demographic profiles, histories of cigarette smoking, results of pulmonary function tests, and the pathologic, clinical and therapeutic characteristics of the cancers were retrospectively retrieved from their medical records. The clinical stage of the cancer was based on the UICC-TNM classification version 7. Written informed consent was obtained from all patients and volunteers. This study was approved by the institutional review board of Tokai University School of Medicine (#09R-044).

### Measurements of VOC in exhaled air

We recently developed a double cold-trap method to remove VOC from the atmosphere and concentrate VOC in the exhaled air [7]. Briefly, the study participants inspired purified air synthesized through a cold purification tube, and expired into a sampling bag. VOC in the expired gas are trapped in a cold concentration tube, then released by heating to 80°C for 30 min. The concentrated, expired gas was sampled with a syringe and analyzed with a GC-2014 gas chromatograph (Shimadzu Co., Kyoto, Japan) equipped with a G-100, open-tubular, wide-bore, no-polarity column (Chemicals Evaluation and Research Institute, Japan) and a FID-2014 flame ionization detector (Shimadzu Co., Japan). The acquired GC profile was analyzed with dedicated software (GC solution Ver.2, Shimadzu Co., Japan). The lower limits of detection were 0.048 ppb for nonane, 0.050 ppb for octane, 0.054 ppb for heptane, 0.060 ppb for cyclohexane, 0.064 ppb for toluene, 0.067 ppb for xylene, 0.068 ppb for ethanol, acetone and phenyl acetate, 0.069 ppb for ethyl benzene, 0.074 ppb for hexane and benzene, 0.098 ppb for isoprene and 0.099 ppb for decane.

### Statistical analysis

The data are presented as medians and ranges. Measurements below the detection limits were substituted as half the concentration of the lower limit of detection. The concentrations of VOC in exhaled air of healthy volunteers versus patients with lung cancer were compared, using the adjusted Mann-Whitney U-test. The differences in VOC between the two populations, which were statistically significant after Bonferroni’s correction, were further analyzed. The relationship between concentrations of VOC and history of smoking, results of pulmonary function tests, histology and clinical stage of lung cancer were examined with Kruskal-Wallis test and Wilcoxon’s rank test. The trends in VOC concentrations according to the clinical stages of the disease were analyzed with Jonckheere-Terpstra trend test. A receiver operating characteristic (ROC) curve was constructed to examine the sensitivity and specificity of exhaled VOC as a biomarker of lung cancer. The statistical analyses were performed, using the IBM SPSS statistics ver. 21 (IBM, Chicago, IL) and Graphpad prism Ver. 5 (Graphpad software Inc., San Diego). The results were two-sided, and a *p* value < 0.05 was considered statistically significant.

## Results

We enrolled 37 volunteers (20 women) between the ages of 24 and 64 years (controls), and 116 patients with lung cancer (88 men) between the ages of 36 and 96 years in this study (Table 1).

**Table 1:** Characteristics of healthy volunteers and patients with Small Cell (SCLC) and Non-Small Cell Lung Cancer (NSCLC).

	Healthy subjects	Patients		
	n = 37	All	NSCLC	SCLC
		n = 116	n = 91	n = 25
Age	27 (24–64)	66 (36–96)	65 (36–96)	69 (45–85)
Men	17 (46)	88 (76)	68 (75)	20 (80)
Smoking status				
Non-smoker	30 (81)	23 (20)	22 (24)	1 (0)
Former smoker	5 (14)	51 (44)	40 (44)	11 (44)
Current smoker	2 (5)	42 (36)	29 (32)	13 (52)
Pack-years^1^	0 (0–10)	40 (82–150)	40 (2–138)	53 (25–150)
Pulmonary function test results	NA	n = 88	n = 69	n = 19
FEV_1_/FVC (%)^1^		70 (37–98)	70 (37–98)	70 (43–82)
FEV_1_/FVC < 0.7		47 (53)	37 (54)	10 (52)
FEV_1_, %predicted^1^		72 (29–134)	74 (29–104)	70 (32–134)
Histology of cancers				
Small cell lung cancer		25 (22)		
Squamous cell carcinoma	NA	18 (16)		
Adenocarcinoma		55 (46)		
Others* / non-small cell lung cancer		18 (16)		
Clinical stages of lung cancer				
I A & B		9 (8)	5	4
II A & B	NA	2 (2)	0	2
III A		23 (20)	17	6
III B		24 (21)	19	5
IV		58 (50)	50	8
Prior cancer therapy				
None		44 (38)	35 (38)	9 (4)
Chemotherapy	NA	53 (46)	42 (46)	11 (44)
Radiotherapy		9 (8)	9 (10)	0 (0)
Chemoradiotherapy		10 (9)	5 (5)	5 (2)

Values are medians (ranges) or numbers (%) of observations. NA = not applicable;

*including large cell neuroendocrine carcinoma and NSCLC not otherwise-specified

The patients with lungs cancer were significantly older (*p* < 0.03) and more likely to be men (*p* < 0.001) than the controls. The distribution of the various histological types of lung cancers was similar to that previously reported by other Japanese institutions, except for a slightly higher proportion (22%) of small cell lung cancers (SCLC) [8,9]. Most patients had non-resectable disease, in clinical stages IIIA (19%), IIIB (21%), or IV (48%).

We compared the concentrations of 14 different VOC contained in exhaled air between controls and patients with lung cancer, using a *p* value < 0.003 to correct multiple comparisons (Table 2).

**Table 2:** Exhaled VOC concentrations in studied groups.

	Healthy subjects (n = 37)		Patients with lung cancer (n = 116)		*p*
Ethanol	13.5	(0.00–68.7)	14.5	(0.00–160)	0.45
Acetone	17.5	(0.00–68.7)	19.6	(0.00–1418)	0.43
Isoprene	31.5	(3.76–184)	43.4	(2.88–202)	0.02
Hexane	0.25	(0.00–4.82)	0.25	(0.00–6.28)	0.47
Benzene	0.10	(0.00–0.48)	0.20	(0.00–0.81)	0.42
Cyclohexane	0.10	(0.00–0.48)	0.20	(0.00–1.71)	0.002**
Heptane	0.15	(0.00–6.26)	0.17	(0.00–7.36)	0.69
Toluene	0.19	(0.00–1.13)	0.26	(0.00–1.82)	0.02
Octane	0.12	(0.00–3.66)	0.16	(0.00–3.38)	0.32
Ethyl benzene	0.10	(0.00–1.33)	0.15	(0.00–3.41)	0.02
Xylene	0.07	(0.00–1.40)	0.16	(0.00–5.60)	0.0001**
Nonane	0.07	(0.00–1.35)	0.10	(0.00–18.0)	0.01
Decane	2.88	(0.26–18.5)	4.25	(0.06–62.9)	0.31
Phenyl acetate	0.78	(0.01–4.27)	0.53	(0.00–7.53)	0.46

Values are median ppb (range);

**p < 0.003

**Fig 1:**
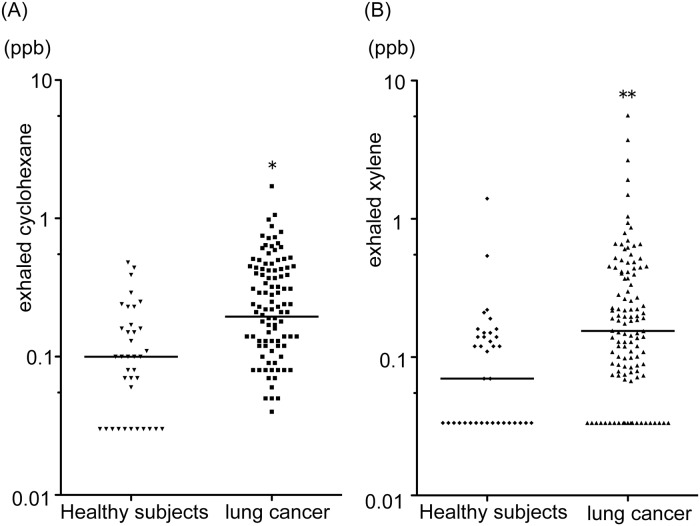
Exhaled cyclohexane (A) and xylene (B) concentrations in healthy subjects and patients with lung cancer. The horizontal lines indicate the median values. *p = 0.002, **p = 0.0001 versus healthy subjects.

**Fig 2:**
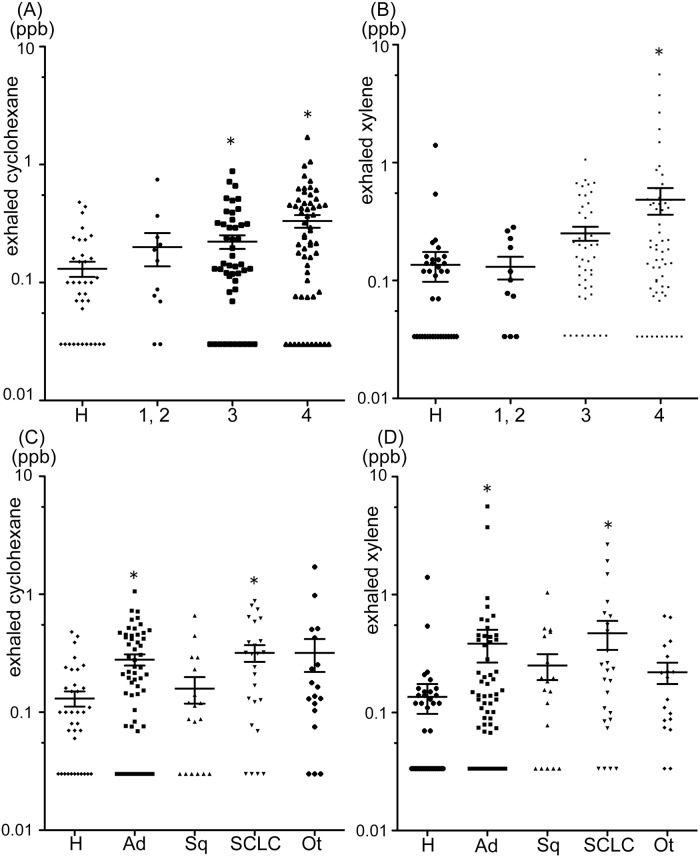
Relationships between exhaled cyclohexane (A) and xylene (B) and clinical stages of lung cancer and between exhaled cyclohexane (C) and xylene (D) and pathological types of lung cancer. The horizontal lines indicate the median values. *p < 0.05, compared with healthy subjects by Kruskal-Wallis test. H = healthy subjects; 1,2 = clinical stage 1 or 2; 3 = clinical stage 3; 4 = clinical stage 4; Ad = adenocarcinoma; Sq = squamous cell carcinoma; SCLC = small cell carcinoma; Ot = others, including large cell neuroendocrine carcinoma and NSCLC not otherwise specified.

The concentrations of cyclohexane and xylene in the exhaled gas of patients with lung cancer were significantly higher (*p* = 0.002 and *p* < 0.0001, respectively) than in the control group (Table 2; Fig 1). Furthermore, the concentrations of these VOC increased significantly (each *p* < 0.001) as the clinical stage of lung cancer advanced (Fig 2A and 2B). The concentrations of cyclohexane in the gas expired by patients with stage IV disease and those of xylene in the gas expired by patients with stages III and IV disease were significantly higher than in the gas expired by the controls; however, the concentrations of these VOC in controls and in patients with early-stage lung cancer were similar (Fig 2C and 2D).

When we examined the relationship between the histological types of lung cancer and the concentrations of cyclohexane and xylene, we found that these concentrations were significantly higher in patients with adenocarcinoma or SCLC than in controls, though the difference between controls and patients with squamous cell carcinoma was less marked (Fig 2C and 2D). The differences among histological types did not reach statistical significance.

**Fig 3:**
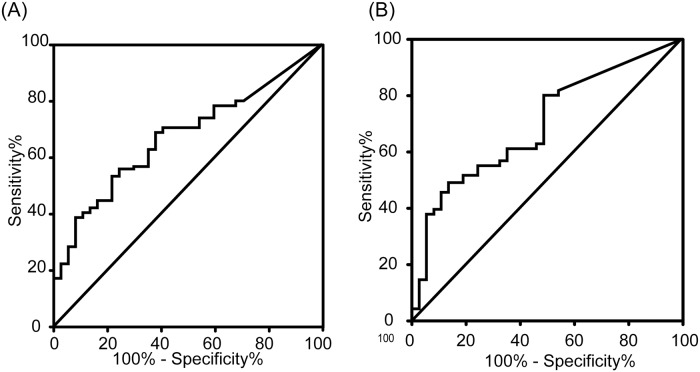
Identification of patients with lung cancer by cyclohexane (A) and xylene (B) ROC analysis.

To determine whether factors other than the presence and clinical stages of lung cancer might influence the concentrations of exhaled VOC, we examined the relationship between their concentrations in the breath samples obtained from patients and putative confounding factors, including age, gender, smoking status, and pulmonary function, though found no relationship between the concentrations of cyclohexane or xylene and these variables (S1 and S2 Tables). The ROC curve constructed to examine the reliability of exhaled VOC as a biomarker of lung cancer revealed an area under the curve of 0.67 for cyclohexane and 0.71 for xylene (Fig 3). The sensitivity and specificity for the diagnosis of lung cancer, using cyclohexane, were 0.53 and 0.78, respectively, with a cut-off value of 0.174 ppb, and 0.49 and 0.86, respectively, using xylene, with a cut-off value of 0.167 ppb. We also analyzed the ability of combining these two VOC to identify lung cancer. The sensitivity and specificity in the detection of lung cancer were 0.75 and 0.78, respectively, if either cyclohexene was > 0.174 ppb or xylene was > 0.167 ppb. Importantly, the composite diagnosis based on exhaled concentrations of cyclohexene and xylene yielded a sensitivity and specificity of 0.73 and 0.78 for stages I and II diseases.

**Fig 4:**
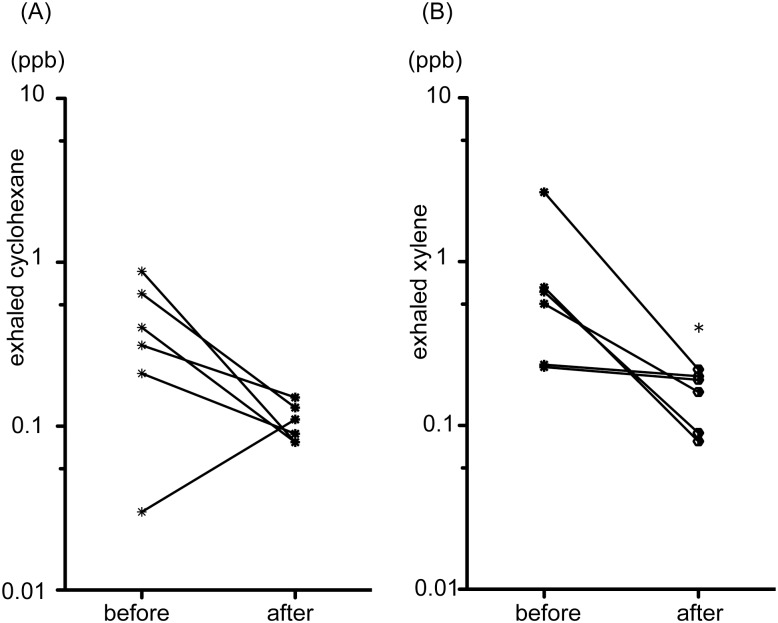
Comparison between exhaled cyclohexane (A) and xylene (B) in 6 patients with small cell lung cancer before treatment and after response to therapy. *p < 0.05 versus before treatment.

In 6 patients whose SCLC responded partially to chemotherapy or chemoradiotherapy (S3 Table), the mean concentration of xylene decreased significantly after treatment (*p* < 0.05; Fig 4). Likewise, the concentration of cyclohexane tended to decrease after the treatments (*p* = 0.06; Fig 4).

## Discussion

We used a new, double cold-trap method to measure very low concentrations of VOC in the expired gas without being contaminated by ambient VOC, and identified cyclohexane and xylene as possible biomarkers of lung cancer, especially in advanced stages.

Xylene, which belongs structurally to the group of aromatic VOC, is contained in various common household products and cigarette smoke [10,11]. The concentration of xylene is elevated in the exhaled air of patients with lung cancer. However, Poli et al. argued that these high concentrations in patients presenting with non-small cell lung cancers (NSCLC) are due to smoking and chronic obstructive disease [12]. In the present study, however, we found that the exhalation of xylene was increased independently of the smoking status or the presence of airflow limitation, though was closely related to the progression of lung cancer. An important difference between the study by Poli, et al. and ours is the histology and stage of lung cancer and the methods used to measure VOC in exhaled air. In contrast to that study, which examined only resectable NSCLC, the majority of our patients presented with non-resectable lung cancers, including SCLC [12].

This is, to the best of our knowledge, the first report of an increase in the concentrations of cyclohexane, one of the cycloalkanes, in the exhaled air of patients with lung cancers. Although there may be a background content of cyclohexane from gasoline vapors in the ambient air [13], our observations showed an unequivocal relationship between the concentration of this gas in exhaled air and the progression of lung cancer. Furthermore, we present evidence, albeit preliminary, of a decrease in the concentrations of cyclohexane after successful treatment of SCLC.

One of the objectives of this study was to identify a VOC that could be measured to detect lung cancer in an early stage. However, none of the 14 VOC we measured seemed to be a promising biomarker of early disease. The combined measurement of cyclohexane and xylene may increase the sensitivity of early cancer detection as previously demonstrated in the studies using eNose or colorimetric sensor array [14,15], a hypothesis we could not validate definitively because of the small number of patients presenting with early-stage cancer. The concentrations of cyclohexane and xylene increased as the lung cancers progressed, and decreased following successful treatments of 6 patients, suggesting that these VOC could monitor the progression of SCLC and NSCLC as well as the outcome of treatment. This is the first report of a relationship between clinical stages of lung cancer and concentrations of exhaled VOC, a relationship that was not confirmed previously [16–18].

There are several limitations in our study. First, our study included patients who were already on chemotherapy for lung cancer at the time of enrollment, which might have significantly increased the concentrations of exhaled VOC. However, in a subgroup of 44 patients not previously treated for lung cancer, the concentrations of exhaled cylohexane (*p* = 0.011) and xylene (*p* = 0.003) were also significantly higher than in controls. Second, the age and smoking history in controls versus patients with lung cancer were not closely matched, although neither the age nor the smoking status had a significant influence on the concentration of exhaled VOC in the patients with lung cancer. Third, the present study, designed as an exploratory research, was underpowered to identify VOCs useful for early diagnosis of lung cancer due to small number of early-stage cases. Future study focusing on early-stage lung cancer with appropriate sample size calculation would be required. A prospective study is also needed to prove the usefulness of exhaled VOC measurement to evaluate treatment effects.

In conclusion, measurement by the double cold-trap method of the exhaled concentrations of VOCs is a non-invasive tool that may be useful to diagnose lung cancer as well as monitor its progression and regression.

## Supporting information

S1 Table Comparison of VOC between groups divided by confounding variables. (DOCX)

S2 Table Correlation between confounding variables and exhaled VOC. (DOCX)

S3 Table Characteristics of 6 patients with small cell lung cancer who responded partially to treatment. (DOCX)
