# Supplementary material for: Clinical contributions of exhaled volatile organic compounds in the diagnosis of lung cancer
Source: PLoS One. 2017 Apr 6;12(4):e0174802. doi: 10.1371/journal.pone.0174802 (PMC5383041; doi:10.1371/journal.pone.0174802)
Supplement: S1 Table — (DOCX) [file pone.0174802.s001.docx]

**Supplemental table 1. Comparison of VOC between groups divided by** confounding variables

|  | **Gender** | | | |  |
| --- | --- | --- | --- | --- | --- |
|  | **Men** | **Women** | | | ***p**** |
| **Cyclohexane** | 0.19 (0.03-1.71) | 0.21 (0.03-1.06) | | | 0.882 |
| **Xylene** | 0.18 (0.03-5.60) | 0.13 (0.03-0.94) | | | 0.214 |
|  | **Smoking** | | | |  |
|  | **Non-smoker**  **(n=23)** | **Former smokers**  **(n=51)** | **Current smokers**  **(n=42)** | ***P***** | ***P****** |
| **Cyclohexane^1^** | 0.24 (0.03-1.06) | 0.19 (0.03-0.80) | 0.17 (0.03-1.71) | 0.775 | 0.496 |
| **Xylene^1^** | 0.14 (0.03-5.60) | 0.14 (0.03-3.72) | 0.21 (0.03-2.66) | 0.213 | 0.204 |
|  | **Pulmonary function** | | | |  |
|  | **FEV_1_/FVC > 0.7**  **(n=41)** | **FEV_1_/FVC < 0.7**  **(n=47)** | | ***P***** | ***P****** |
| **Cyclohexane^1^** | 0.29 (0.03-1.71) | 0.17 (0.03-0.98) | | 0.269 | 0.152 |
| **Xylene^1^** | 0.19 (0.03-5.60) | 0.15 (0.03-3.72) | | 0.728 | 0.456 |

Values are medians (ranges); *Mann-Whitney U-test; **Kruskal-Wallis test, ^***^Jonckheere-Terpstra trend test among 3 groups.
