# Supplementary material for: Clinical contributions of exhaled volatile organic compounds in the diagnosis of lung cancer
Source: PLoS One. 2017 Apr 6;12(4):e0174802. doi: 10.1371/journal.pone.0174802 (PMC5383041; doi:10.1371/journal.pone.0174802)
Supplement: S2 Table — (DOCX) [file pone.0174802.s002.docx]

**Supplemental table 2. Correlation between** confounding variables and exhaled **VOC**

|  | **Cyclohexane** | **Xylene** |
| --- | --- | --- |
| **Age** |  |  |
| **ρ*** | -0.138 | -0.080 |
| ***p***** | 0.139 | 0.391 |
| **Smoking (pack-year)** |  |  |
| **ρ*** | 0.011 | 0.251 |
| ***p***** | 0.903 | 0.267 |
| **FEV_1_, % predicted** |  |  |
| **ρ*** | 0.095 | 0.052 |
| ***p***** | 0.378 | 0.633 |

*spearman’s rank correlation coefficient between VOC and confounding factors;

**correlation between VOC and confounding factors by Spearman’s rho test.
