# Supplementary material for: Clinical contributions of exhaled volatile organic compounds in the diagnosis of lung cancer
Source: PLoS One. 2017 Apr 6;12(4):e0174802. doi: 10.1371/journal.pone.0174802 (PMC5383041; doi:10.1371/journal.pone.0174802)
Supplement: S3 Table — (DOCX) [file pone.0174802.s003.docx]

**Supplemental table 3. Characteristics of 6 patients with small cell lung cancer who responded partially to treatment**

| Age | 66（45-74） |
| --- | --- |
| Men | 5 |
| Smoking status  Non-smoker  Former smoker  Current smoker  Pack-years | 0  3  3  56 (41-135) |
| Pulmonary function test results  FEV1/FVC (%)  FEV1, %predicted | (n = 4)  72 (65-80)  76 (54-97) |
| Clinical stages of lung cancer  I A & B  II A & B  III A & B  IV | 1  0  4  1 |
| Lung cancer therapy  Chemotherapy  Chemoradiotherapy  Surgical resection and chemotherapy | 2  3  1 |
| Months between measurements | 6 (3-7) |

Values are medians (ranges) or numbers of observations
